# Intermolecular Electrostatic Interactions in Cytochrome *c* Protein Monolayer on Montmorillonite Alumosilicate Surface: A Positive Cooperative Effect

**DOI:** 10.3390/ijms25136834

**Published:** 2024-06-21

**Authors:** Svetlana H. Hristova, Alexandar M. Zhivkov

**Affiliations:** 1 Department of Medical Physics and Biophysics, Medical Faculty, Medical University—Sofia, Zdrave Str. 2, 1431 Sofia, Bulgaria; 2 Scientific Research Center, “St. Kliment Ohridski” Sofia University, 8 Dragan Tsankov Blvd., 1164 Sofia, Bulgaria

**Keywords:** cytochrome *c*, montmorillonite, protein electrostatics, macromolecular docking, cooperative effect, surface macromolecular aggregation

## Abstract

Montmorillonite (MM) crystal nanoplates acquire anticancer properties when coated with the mitochondrial protein cytochrome *c* (cytC) due to the cancer cells’ capability to phagocytize cytC-MM colloid particles. The introduced exogenous cytC initiates apoptosis: an irreversible cascade of biochemical reactions leading to cell death. In the present research, we investigate the organization of the cytC layer on the MM surface by employing physicochemical and computer methods—microelectrophoresis, static, and electric light scattering—to study cytC adsorption on the MM surface, and protein electrostatics and docking to calculate the local electric potential and Gibbs free energy of interacting protein globules. The found protein concentration dependence of the adsorbed cytC quantity is nonlinear, manifesting a positive cooperative effect that emerges when the adsorbed cytC globules occupy more than one-third of the MM surface. Computer analysis reveals that the cooperative effect is caused by the formation of protein associates in which the cytC globules are oriented with oppositely charged surfaces. The formation of dimers and trimers is accompanied by a strong reduction in the electrostatic component of the Gibbs free energy of protein association, while the van der Waals component plays a secondary role.

## 1. Introduction

Cancer is one of the two most socially important diseases leading to premature death, alongside cardiovascular ones. The number of new cancer cases increases by 3–5% per year in the world [1,2]. However, the modern resources are insufficient for the successful treatment of the diversity of cancer diseases. This requires the development of new approaches in chemo-, immuno-, and radiotherapy. Anticancer chemotherapy has several disadvantages, with the primary one being its unselective effect on both cancerous and normal dividing cells. An alternative approach is based on the activation of apoptosis [3]: a mechanism of damaged cell suicide in healthy macroorganisms, which functions as a cascade of enzyme reactions finishing with complete destruction of the cell [4].

### 1.1. Apoptosis-Inducing Colloid Particles

Apoptosis can be activated by introduction of exogenous cytochrome *c* (cytC): a water soluble globular hemoproteid that normally functions as an electron carrier in the electron transport protein complex located in the internal mitochondrial membrane [5]. However, cytC cannot penetrate trough the cytoplasmic membrane of biological cells. Therefore, to transport cytC into the cancer cells, their property to absorb extracellular particles by phagocytosis (an uptake that begins with adsorption and envelopment of the particle by the cytoplasmic membrane) can be used. This mechanism limits the size range of the particles; the optimal size is about half a micrometer. Since normal cells cannot phagocytize, except those of the immune system, the cytC-bearing particles can predominantly be introduced into cancer cells, and this provides the selectivity of the anticancer effect, in contrast to anticancer chemotherapeutics.

For the cytC-bearing particles, crystal nanoplates of 1 nm thickness are used, obtained from the clay mineral montmorillonite (MM) by cleavage of its colloid particles, normally appearing as packs of monoplates. To meet the size condition optimal for phagocytosis, the MM suspension is separated to obtain a fraction of half-micrometric size. Due to their plate-like form and the extremely high surface/thickness radio, MM nanoplates can adsorb a larger number of cytC globules per unit area at a surface/mass ratio equal to that of spherical particles. The adsorption is electrostatically conditioned because the MM nanoplates are negatively charged, regardless of the pH, and the cytC globules are positively charged under pH 9 [6,7]. At saturated adsorption, cytC globules form a protein monolayer with a density of eight globules per 100 nm^2^, which occupies two-thirds of the MM surface [8]. Using an aqueous suspension of cytC-MM hybrid particles, we achieved 97% cytotoxicity in an in vitro experiment with a colon cancer cell culture [9].

### 1.2. Purpose of the Investigation

To achieve the apoptosis threshold with the uptake of a smaller number of cytC-MM hybrid particles, it is necessary to adsorb more cytC macromolecules onto every MM monoplate. However, the surface area of the MM monoplates is limited by the maximal size of the particles appropriate for phagocytosis. The degree of completed adsorption is a function of the protein concentration in the suspension and the adsorption capability of the particle surface. Therefore, to introduce a higher number of cytC globules with the uptake of one hybrid particle by a cancer cell, it is necessary to reach the maximal density of the protein monolayer at saturated adsorption. The protein density in the surface layer depends on the geometric and electric properties of the protein globules (the size, charge, and charge asymmetric distribution), and the intermolecular interactions between the neighboring globules. However, the organization of cytC globules in the protein monolayer remains unknown; this is the object of the present research. This investigation aims to disclose whether cytC globules are single, or whether they form associates (complexes) at their surface concentration caused by the adsorption on MM nanoplates. For this purpose, we investigate the intermolecular interactions at an increasing degree of occupation of the nanoplate surface employing a set of methods: physicochemical (microelectrophoresis, and static and electric light scattering) and computer (protein electrostatics and docking).

### 1.3. Research Approach

To disclose whether the adsorbed cytC globules are adsorbed independently or interact, we experimentally investigate the adsorption isotherm: the amount of adsorbed protein at a fixed temperature as a function of the protein concentration in the suspension. In the case of protein–protein interactions, a cooperative effect should appear: a negative one (caused by the predominance of the electrostatic repulsion over the van der Waals attraction), or a positive one in the opposite case. The adsorption isotherm can reveal the type of intermolecular interaction: the curve should be bent downward at predominant repulsion, bent upward at predominant attraction, or linear in the absence of intermolecular interactions. We investigated the steady-state adsorption of cytC in an MM suspension by employing the method of static light scattering, which allows for the measurement of MM nanoplate mass growth under protein adsorption.

To estimate the probability of cytC globule association, as a first step, we calculated the electrostatic potential on a single cytC globule employing a computer program for protein electrostatics. Then, using a program for protein docking, we generated models of cytC dimers and trimers in which the cytC globules are oriented according to their geometrical surface and local electrostatic potential. To estimate the thermodynamical stability of the generated models, we calculated the association energy Δ*G*_assoc_ as a measure of Gibbs free energy decrease at protein aggregation. The Δ*G*_assoc_ quantity was determined by the intermolecular interactions: electrostatic repulsion and van der Waals attraction, which cause the protein association at increasing of cytC concentration on the MM nanoplate surface. The combination of physicochemical and computer techniques allows for us to explain the found nonlinearity of the adsorption isotherm by the emergence of protein associates in which cytC globules are oriented by oppositely charged surfaces with negative to positive local potentials.

## 2. Results and Interpretation

The net charge of globular proteins is specifically determined by the type, content, and sequence of the amino acid residues. It also depends on the 3D folding of the polypeptide chain. The surface charge density (the number of Coulombic charges per unit surface) of cytC-MM hybrid particles is a function of the pH-dependent net charge of cytC macromolecules and the density of the protein layer (the number of cytC globules per unit area of MM surface). Both factors determine the interactions between the adsorbed protein globules.

### 2.1. Net Charge and Electrostatic Potential of a Single cytC Globule

**Figure 1:**
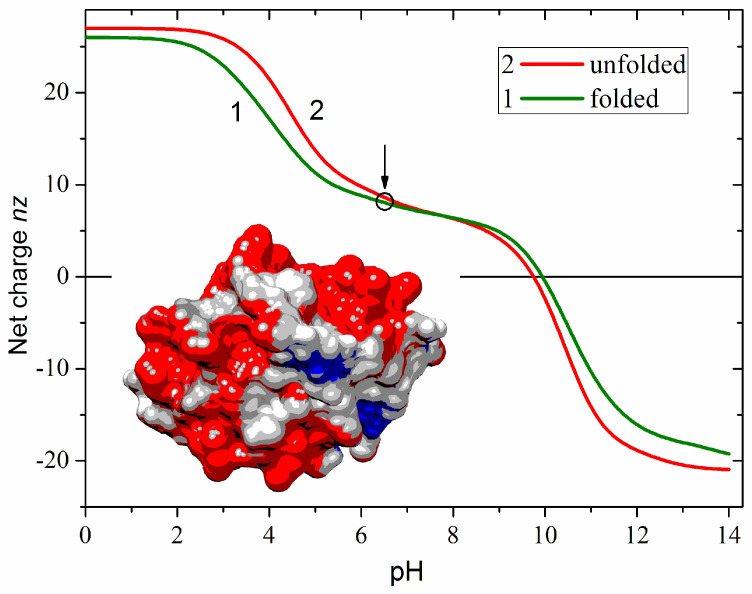
Computed pH dependence of the net charge *nz* of equine cytC apoprotein in aqueous medium in 3D folded (curve 1) and unfolded (curve 2) conformations of the polypeptide chain. The circle and arrow denote pH 6.5 used in the experiments. Insert: Cytochrome *c* (cytC) globule [PDB: 1HRC] at pH 6.5 colored according to the electrostatic potential on its surface: positive (red), negative (blue), and almost neutral (gray).

Figure 1 shows the net charge *nz* of a free cytC apoprotein in two conformations of the polypeptide chain: globular (native with fixed 3D atomic coordinates) and unfolded (fully denatured, random coil). The *zn* is defined as the algebraic difference between the positive (+*zn*^+^) and negative (–*zn*^−^) Coulombic charges emerging in an aqueous medium as a result of ionization of the chargeable groups of the amino acid residues: protonation of the amino group (–NH^2^ ↔ –NH^3+^) of lysine, guanidine group of arginine and imidazole group of histidine, or deprotonation (–COOH ↔ –COO^−^) of the carboxylic groups of aspartic and glutamic acids. At neutral pH, the net charge of cytC is positive due to the predominance of the protonated over the deprotonated groups. The *nz* (pH) dependence suggests that pH 6.5 as the most suitable for adsorption of cytC globules on the negatively charged MM nanoplates because at this pH the cytC macromolecules are conformationally stable and their positive net charge is almost pH-independent (the quasi-plateau of the *nz* (pH) curve in the range pH 6–9). The difference in the two *nz* (pH) curves is caused by the irregular localization of the Coulombic charges on the surface of the protein globule (determined by the amino acid sequence and 3D conformation of the polypeptide chain). This results in either a positive or negative local electrostatic potential on the globule surface, which leads to a decreased or increased local proton concentration in the surrounding aqueous medium. The difference between the local and bulk pH leads to different values of the apparent dissociation constants pK_a_ of the chargeable groups in folded and unfolded conformations of the polypeptide chain.

**Figure 2:**
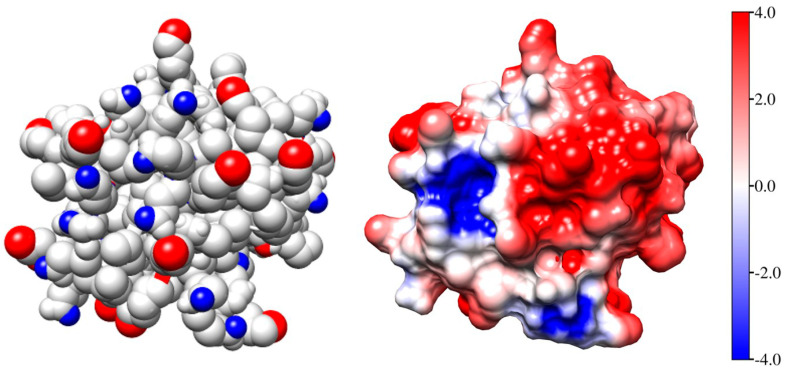
The Coulomb charges (**left** model) and the surface electrostatic potential (the **right** model) of identically oriented equine cytochrome *c* [PDB: 1HRC] globules at pH 6.5. The atoms are colored in red (positive nitrogen atoms in protonated amino groups –NH^3+^), in blue (the negative oxygen O^−^ atoms in deprotonated carboxylic groups –COO^−^), or in white (the uncharged atoms). The electrostatic potential on the surface of cytC globule (the right model) is visualized in the range *kT*/*e* = ± 4 J/C = 4 × 26.7 mV at 37 °C (the scale on the right), according to its value and sign: positive (red), negative (blue), or neutral (white).

Figure 2 (the left model) shows the Coulombic charges of equine cytochrome *c* (cytC) in the 3D conformation of its polypeptide chain (Section 3.3) at pH 6.5: the positively charged nitrogen atoms (colored in red) in the protonated amino (NH^3+^) and guanidine groups of the lysine and arginine amino acid residues, and the negatively charged oxygen atoms (colored in blue) in the dissociated carboxylic COO^−^ groups of aspartic and glutamic amino acid residues. Every colored atom bears one Coulombic charge obtained as a result of association or dissociation of proton H^+^ (which is borne by the protonated water molecules H_3_O^+^ in the medium): protonation (NH_2_ → NH^3+^) of the amino groups, or deprotonation (COOH → COO^−^) of the carboxylic groups.

The right model in Figure 2 and the insert in Figure 1 show the electrostatic potential on the surface of the cytC globule at pH 6.5, computed by a program for protein electrostatic (Section 4.4). The potential is created by the Coulombic charges of the protonated (positively charged) nitrogen atoms and the oxygen atoms bearing extra electron (negatively charged) (the left model in Figure 2). The local electrostatic potential is computed taking into account the atomic coordinates of the Coulombic charges and the individual dissociation constants K_a_ of all chargeable groups in the cytC macromolecule in 3D (native) conformation of its polypeptide chain. The local potential is visualized by coloration according to its value and sign in red (positive), blue (negative), and white (neutral) in the range *kT*/*e* = ±4 J/C (the scale on the right), where *k*—Boltzmann constant [J/K]; *T*—absolute temperature [K]; *e*—charge of the proton [C]; 1 *kT*/*e* [J/C] = 26.7 mV at 37 °C.

### 2.2. Electrophoretic Mobility of MM and cytC-MM Nanoplates

**Figure 3:**
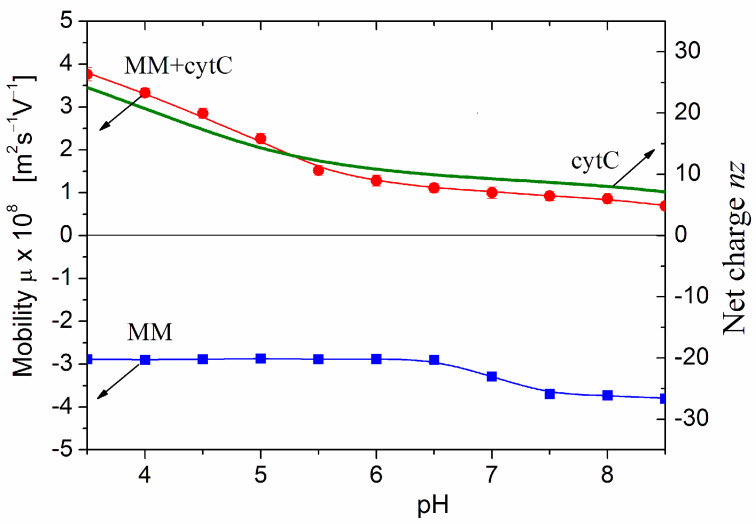
pH-dependences of computed net charge *nz* of free cytC globule (smooth curve, the right ordinate) and the measured electrophoretic mobilities μ (the left ordinate) of bare MM plates and cytC-MM hybrid particles at saturated protein adsorption (10:3 mg/mg cytC/MM) at constant ionic strength. The arrows point to the corresponding ordinates for the *nz* and μ curves.

Figure 3 presents pH dependencies of the electrophoretic mobility μ of bare MM nanoplates and cytC-MM hybrid particles at saturated protein adsorption. The negative sign of μ means that the MM nanoplates migrate to the anode in a direct electric field. Therefore, their surface electric potential is negative, being created predominately by negative Coulombic charges. The μ(pH) curve is almost pH-independent; this fact discloses that the charges are located into the crystal structure of MM nanoplate (Section 3.6). The small step in the μ(pH) curve at pH 7.0 is caused by adsorption/desorption of H_3_O^+^ (hydroxonium cations) and OH^−^ (hydroxyl anions) on the edges of MM nanoplates, where hydrated metal oxide groups are located [10]. At pH < 6.5, the adsorbed cations diminish the negative net charge of MM nanoplate or increase it at pH > 7.5 because of replacement of the cations by anions. However, this pH-dependent charge does not influence the adsorption because the three nanometric cytC globules are too small in comparison with the half-micrometric MM nanoplates and are three times bigger than the 1 nm thickness of MM monoplate. Due to this difference in the sizes, the protein globules are adsorbed on the surface but not on the edges of the MM nanoplates.

The positive sign of the electrokinetic potential μ of cytC-MM hybrid nanoplates (Figure 3), indicating that the particles migrate to the cathode, means that at pH 6.5 (the arrow in Figure 1), the total positive charge of cytC globules is high enough to recharge the negative MM surface at saturated protein adsorption. I.e., the electrostatic adsorption of the positively charged cytC globules on the negatively charged MM nanoplates is over-equivalent. The comparison shows that the pH curves of the electrophoretic mobility μ (pH) of cytC-MM particles and the net charge *nz* (pH) of free cytC macromolecules have near courses (Figure 3). This fact reveals that: (a) the negative electrostatic potential of MM surface is almost fully shielded by the adsorbed cytC globules (otherwise, the slope of the MM + cytC curve should be less steep in comparison with cytC curve in the range pH 3.5–5.5 because of the horizontal course of the MM line), and (b) the pH behavior of cytC globules is the same whether being free in the bulk or adsorbed on the dielectric MM surface.

The main inference obtained from the results in Figure 3 is that the surface electrostatic potential of cytC-MM particles (at saturated protein adsorption) is positive and remains unaltered in the range pH 6.8–7.6 where cancer cells maintain their high vitality. The positive potential means that the adsorption of cytC-MM particles on the negatively charged cytoplasmic membrane of the cancer cells is electrostatically driven.

### 2.3. Size and Mass of cytC-MM Nanoparticles

As an indicator for adsorption of cytC globules on MM nanoplates, we employ the methods of static light scattering and electrophoretic mobility of colloid particles in aqueous suspension, which are based on different physical quantities: the particle mass and the surface electric potential (Section 4.2 and Section 4.3). The mass could grow as a result of adsorption of cytC globules onto MM nanoplates and/or aggregation of cytC-MM plates. To analyze the protein adsorption, it is necessary to exclude the particle aggregation when measuring the light scattering coefficient *R*_θ_. Then, the protein concentration dependence *M*(*C*_cyt_) of the mass *M* of cytC-MM particles on the *C*_cyt_ concentration provides information about the adsorption. Earlier, we found suitable weight concentrations at which aggregation does not emerge: *c* = 3 mg/L MM and *C*_cyt_ = 0–3 mg/L or 10–100 mg/L cytC in aqueous suspension at pH 6; a strong aggregation occurs at *c/C*_cyt_ = 3:5 mg/mg concentration ratio where a recharging from negative to positive total charge appears [11]. In the two protein concentration ranges of suspension stability, the predominant charge of the cytC-MM nanoplates is negative (at low *C*_cyt_) or positive (at high *C*_cyt_), and this leads to electrostatic repulsion between neighboring plates, which counteract their aggregation under the action of van der Waals attractive forces.

**Figure 4:**
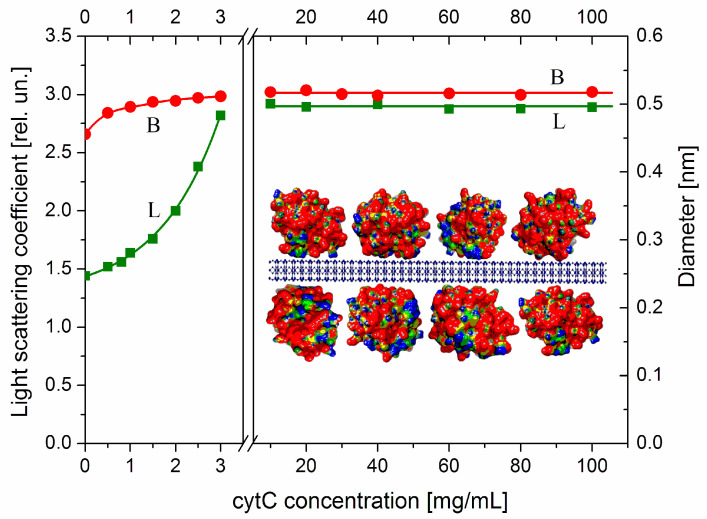
Protein concentration dependences of the effective diameter *B* of cytC-MM particles (curve B, **right** ordinate) and light scattering coefficient (Rayleigh ratio) *R*_θ_ (in relative units, curve L, **left** ordinate) of aqueous suspension containing 3 mg/L MM nanoplates vs. concentration *C*_cyt_ of cytC in the suspension at pH 6.5 and ionic strength 0.1 mM at low (*C*_cyt_ = 0–3 mg/L, **left** panel) or high (*C*_cyt_ = 10–100 mg/L, **right** panel) protein concentrations. The two curves are interrupted in the range *C*_cyt_ = 3.5–9 mg/L. *Insert*: Fragment of the cytC-MM hybrid particle: MM nanoplate (cross-sections) with thickness 1 nm and four adsorbed cytC globules with diameter 3 nm. The electric potential of cytC [PDB: 1HRC] and MM is colored in red (strong positive), yellow (weak positive), green (neutral), and blue (negative).

As an indicator for aggregation, we use the relaxation time τ (the time for 63% decay of the electrooptical effect after the field is switched off), which is determined by the diameter *B* of disk-like colloid particles as MM nanoplates (Section 4.2). This quantity is very sensitive to aggregation due to the cubic dependence τ~*B*^3^, where B is the effective diameter that increases at face-to-face adhesion of the MM nanoplates. The protein concentration dependence *B*(*C*_cyt_) (Figure 4, the right ordinate) of the effective diameter *B*, calculated by Equation (3), shows that the size of cytC-MM particles does not grow at high cytC concentrations in the range *C*_cyt_ = 10–100 mg/L (the right panel), where the adsorption is saturated as the light scattering indicates (line L). The absence of aggregation in this concentration range is due to the positive total charge of cytC-MM particles, which is determined by the cytC adsorption layer at pH 6.5 (Figure 3). The range 3.5–9.0 mg cytC in 3 mg MM, where the suspension is instable because of recharging at 5 mg/L cytC, is excluded from Figure 4. The small grow of the effective diameter *B* at low cytC concentrations (the beginning of the curve *B* in the left panel of Figure 4) reflects the additional friction on the particle surface/medium interface, which is caused by the presence of 3 nm cytC globules on the smooth surface of MM nanoplates. This perturbs the hydrodynamic flow at disorientation of cytC-MM particles after switching off of the electric field. The almost unaltered size *B* discloses absence of aggregation, and this allows for the explanation of the mass increment Δ*M* as a result of only protein adsorption in both the lower (*C*_cyt_ = 0–3 mg/L) and higher (*C*_cyt_ = 10–100 mg/L) concentration ranges in the 3 mg/L MM suspension.

To measure the degree of adsorption, we used the relative mass increment Δ*M*/*M* of cytC-MM particles, calculated by Equation (1) from the measured light scattering coefficient *R*_θ_ of MM suspension at increasing cytC concentrations *C*_cyt_ (Section 4.2). In both cases of cytC adsorption or aggregation of cytC-MM nanoplates, the mass grows with increment Δ*M* = *M*_cyt_ − *M*_0_, where *M*_cyt_ and *M*_0_ are the masses of the MM nanoplates in the presence and absence of cytC in the suspension, respectively. To analyze the adsorption isotherm Δ*M*/*M*_0_ = *f* (*C*_cyt_), we use the relative mass *M*_cyt_/*M*_0_; then, the relative increment is Δ*M*/*M*_0_ = *M*_cyt_/*M*_0_ − 1.

**Figure 5:**
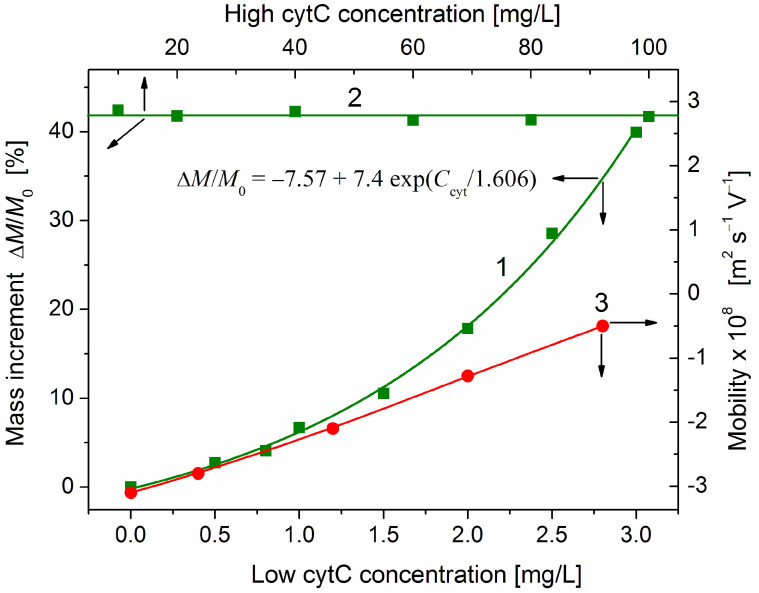
Increment Δ*M*/*M*_0_ of the relative mass of cytC-MM hybrid particles (curve 1 and line 2, left ordinate) and the electrophoretic mobility μ (curve 3, right ordinate) vs. concentration *C*_cyt_ of cytC in two ranges: *C*_cyt_ = 0–3 mg/L (bottom abscissa, curves 1 and 3) and *C*_cyt_ = 10–100 mg/L (top abscissa, line 2) in 3 mg/L MM suspension at pH 6.5 and ionic strength 0.1 mM. The points are experimental data; the fitting curve 1 is calculated according to the shown exponential function. The arrows point to the corresponding abscissae and ordinates.

The protein concentration dependence Δ*M*/*M*_0_ = *f*(*C*_cyt_) of the relative mass increment Δ*M*/*M*_0_ is shown in Figure 5 (curve L). The values of *M*_cyt_ and *M*_0_ are calculated in relative units by Equation (1) from the light scattering coefficient *R*_θ_ (Figure 4), which is measured at completed adsorption (a few minutes after addition of cytC solution to MM suspension is enough to finish the adsorption at all cytC concentrations). In the lowest concentration range (*C*_cyt_ = 0–1 mg/L), the mass *M* of cytC-MM particles grows lineally. This fact discloses that at low surface occupation, the cytC globules are adsorbed independently of each other and the equilibrium constant of adsorption does not depend on the surface filling. The third part of the Δ*M*(*C*_cyt_) dependence (the horizontal line 2) shows out saturated adsorption at *C*_cyt_ ≥ 10 mg/L in 3 mg/L MM suspension. The absence of additional adsorption at this highest protein concentration range is an indication that the cytC globules form a 2D (two-dimensional) protein layer that is completely constructed. Additional cytC globules cannot be adsorbed because of the electrostatic repulsion that an upcoming cytC globule undergoes from the cytC monolayer, both positively charged at pH 6.5 (Figure 3).

At the middle protein concentration range (*C*_cyt_ = 1–3 mg/L), the mass of cytC-MM particles grows exponentially (curve 1 in Figure 5): the relative mass increment increases according to the exponential function Δ*M*_cyt_/*M*_0_~exp(*C*_cyt_/*C*_exp_), where *C*_exp_ is a characteristic cytC concentration at which the exponent is equal to *e* ≈ 2.72. The rising slope of the mass increment curve Δ*M*/*M*_0_ = *f*(*C*_cyt_) indicates a positive cooperative effect. This means that the already adsorbed cytC globules facilitate adsorption of new ones. So, the interactions between the cytC globules on the MM surface are predominantly attractive, although they are positively charged and should undergo electrostatic repulsion. The fact that the noticeable deviation from the linearity appears at *C*_cyt_ ≈ 1.5 mg/L in 3 mg/L MM suspension discloses that the cooperative effect emerges at a ratio of the weight concentrations two times lower than that at which the adsorption reaches saturated values: *C*_cyt_/*c* ≈ 1:1 mg/mg (then, curve 1 reaches line 2).

As an independent indicator for protein adsorption, we use the electrophoretic mobility μ, which decreases (in absolute values) at adsorption of the positively charged (at pH 6.5) cytC globules on the negatively charged MM surface (Figure 3). In this case, the protein concentration dependence μ(*C*_cyt_) is linear (curve 3 in Figure 5). The different courses of the concentration dependences of the mass increment Δ*M*/*M*_0_(*C*_cyt_) and the electrophoretic mobility μ(*C*_cyt_) (curves 1 and 3) disclose that cytC globules form a quasi-monolayer in which a part of cytC globules are associated with the already adsorbed globules but do not lie on the MM surface. The different position of the cytC globules leads to different contributions of the additional cytC globules to the two physical quantities. The mass *M*_cyt_ of cytC-MM nanoplates grows with the adsorbed protein globules independently on their location on the surface. Therefore, the mass increment Δ*M* increases linearly with the number of the adsorbed protein globules. However, the electrophoretic mobility μ is proportional to the occupied/free surface ratio, but not to the adsorbed protein globules because the occupied areas are hydrodynamically shielded independently on the number of the protein globules in the adsorption mono- or multilayer.

### 2.4. Protein-Protein Association

The adsorption isotherm Δ*M*/*M*_0_ = *f*(*C*_cyt_) (the dependence of the relative mass increment on cytC concentration) shows three ranges: linear, exponential, and saturated (curves 1 and 2 in Figure 5). The exponential growth of the mass *M* of cytC-MM particles (the concave part of curve 1) reveals a positive cooperative effect (Section 3.9). Taking into consideration that the deviation from linearity arises at *C*_cyt_ ≈ 1.5 mg/L, which is half the concentration of saturation *C*_cyt_ ≈ 3 mg/L (curve 2 in Figure 5), and that at saturated adsorption, the cytC globules occupy two-thirds of the MM surface [8], it can be inferred that the cooperative effect appears at one-third of the surface occupation. This value (about 33%, found by static light scattering) practically coincides with the 36% surface occupation obtained by AFM measuring of the roughness at adsorption of cytC on mica [12]. At this intermediate degree of surface occupation, the probability increases that an approaching cytC globule will meet already adsorbed ones.

The positive sign of the found cooperative effect (the exponential curve 1 in Figure 5) suggests that the attractive forces (London dispersion and dipole–dipole electrostatic) between two contacting protein globules are stronger than the electrostatic repulsion, which is determined by the positive net charge of cytC globules at pH 6.5 (Figure 3). However, cytC globules do not form associates or crystals in aqueous solution under analogical conditions: protein concentration up to 100 mg/L, pH 6–7 and low ionic strength. An association, as a preliminary step of protein crystallization, is possible only at a very high electrolyte concentration and/or pH near the isoelectric point pI 9.3.

To uncover the mechanism of the cooperative effect, three factors that influence the balance of attractive–repulsive forces between the adsorbed cytC globules must be considered. The first one is the surface-induced concentrating of protein globules at their adsorption on the suspension particles. In this process, the mean distance between the neighboring globules decreases and becomes much shorter than that in protein solution with same concentration. The second factor is the immobilization of the adsorbed protein globules. Then, the attractive forces between a protein globule and the solid surface are stronger than the impacts of the surrounding water molecules, which cause the translational diffusion of the free globules in the bulk of the suspension. The third factor is the inhomogeneity of the electrostatic potential on the surface of cytC globules: positive and negative patches appear because of the irregular disposition of the Coulombic charges of the amino acid residues (Figure 2). An approaching cytC globule, due to its translational and rotational freedom and electrostatic interaction with already adsorbed cytC globules, obtains an orientation at which the two neighboring cytC globules contact with oppositely charged surfaces: negative to positive. In this case, the van der Waals and local electrostatic attractions can predominate over the electrostatic repulsion between the distant charges. When such energetically advantageous mutual orientation is possible, the new cytC globule contacts with globules already adsorbed on the solid surface of MM nanoplate. As a result, the protein globules are not adsorbed singly, but form local associates of a few globules. This electrically induced orientation leads to the formation of a quasi-monolayer of cytC globules at their adsorption on MM surface.

We also considered the possible electrostatic influence of the negatively charged MM plate, which could increase the local concentration of hydroxonium cations H_3_O^+^ (decrease the local pH) and by that change the local electrostatic potential on the surface of cytC globules. This could indirectly influence the protein–protein interactions. However, computer estimations disclosed that the influence of the MM surface on the degree of ionization in the chargeable groups of adsorbed cytC globules is negligible at the chosen pH of 6.5. The reason is that the dissociation constants of the carboxylic and amino groups appear in the acid and basic pH ranges, respectively; an indication of this is the quasi-plateau of *nz* (pH) curve in Figure 1. The role of the negatively charged MM nanoplates is to attract the positively charged cytC globules, that is, the driving force of the electrostatic adsorption. Additionally, being adsorbed on the solid surface, the protein globules undergo van der Waals attraction.

Therefore, it can be concluded that the cooperative effect is caused by the association of cytC globules, which is conditioned by two main factors: (a) the surface-induced concentration and immobilization caused by the electrostatic adsorption, and (b) the irregular distribution of the chargeable amino acid residues, which creates patches of the local electrostatic potential with an opposite sign (positive or negative) on the surface of the cytC protein globules.

### 2.5. Association Energy

**Figure 6:**
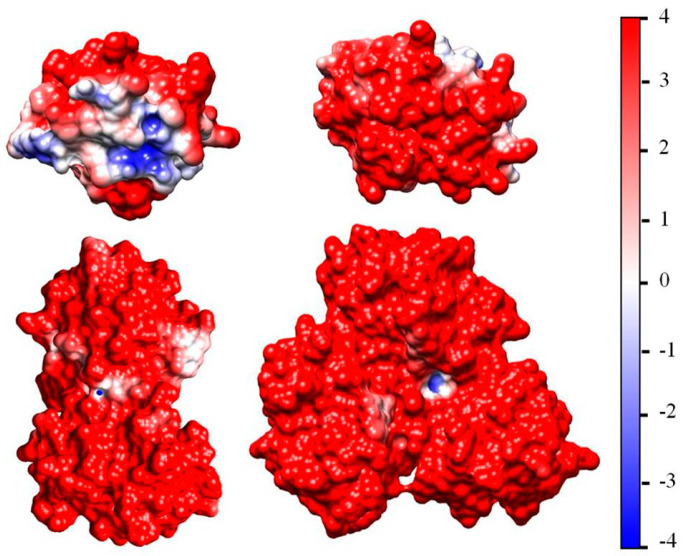
Dimer and trimer (**lower two models**) formed by cytC globules oriented with oppositely charged (negative to positive) contact surface areas (**upper two monomers**). The surfaces of the cytC models [PDB: 1HRC] are colored according to the electrostatic potential (positive—red; negative—blue), computed at pH 6.5, ionic strength 0.0001 mol/L, and temperature 20 °C, and visualized in the range *kT*/*e* = ±4 J/C (the scale on the right).

To quantitatively verify the above hypotheses that the cooperative effect is caused by the association of the cytC globules, we calculated the alteration in free energy upon the formation of protein dimers and trimers. For this purpose, we introduce the term “association energy” Δ*G*_assoc_, defining it as the difference in Gibbs free energy of the protein associate composed by *N* number monomers and their summed-up energy: Δ*G*_assoc_ = Δ*G*_N-mer_ − *N* × Δ*G*_monomer_. The free energy decreases with the formation of thermodynamically stable associates; then, Δ*G*_assoc_ has a negative sign (decrement −Δ*G*_assoc_) and its absolute value |−Δ*G*_assoc_| is a measure for the stability of the protein associate. This dimer and trimer are shown in Figure 6. On the contrary, a positive sign of Δ*G*_assoc_ (increment + Δ*G*_assoc_) means that formation of stable associates is impossible. The computer techniques for the construction of dimers and trimers and the calculation of their association energy are described in Section 3.13, Section 3.14 and Section 3.15.

The results for cytC dimers (*N* = 2) and trimers (*N* = 3) disclose that the sign and value of the association energy Δ*G*_assoc_ strongly depend on the mutual orientation of the protein globules. In the most models, Δ*G*_assoc_ has a positive value (increment + Δ*G*_assoc_), i.e., formation of associates is impossible because the free energy should increase. The reason for this is that in these models, the cytC globules are oriented with identically charged (positive-to-positive) areas of their surfaces, so the electrostatic repulsion does not allow for association. A decrement in the free energy (negative Δ*G*_assoc_) appears when the cytC globules are oriented with surfaces having local electrostatic potential with opposite signs (negative to positive), and the contacting surfaces have complementary reliefs. At such mutual orientation, both components (electrostatic and van der Waals) of the association energy have negative signs and maximal absolute values, i.e., the associate is the most thermodynamically stable.

The association energy of the cytC dimer and trimer with negative-to-positive charged surfaces and different degrees of geometrical complementarity are given in Table 1. The associates dimer-2 and trimer-2, shown in Figure 6, have higher opposite (negative-to-positive) potentials in the contacting surfaces and higher geometrical complementarity; therefore, they are more densely packed and have a smaller size. As a result, they are much more stable in comparison with dimer-1 and trimer-1, as the absolute values |Δ*G*_assoc_| of the association energy disclose. The comparison between the two components of Δ*G*_assoc_ reveals that the electrostatic energy plays the main role in the cytC association, while the contribution of the van der Waals attraction is relatively small. These results quantitatively corroborate the hypothesis that the cooperative effect at adsorption of cytC globules onto MM plates is caused by protein-to-protein electrostatic association.

## 3. Discussion

### 3.1. Chemotherapy

Despite the significant progress in the synthesis of anticancer chemotherapeutics of different classes [13], many unresolved issues still persist. The main drawback is the lack of selectivity, which leads to the destruction of both cancerous and normal dividing cells, including those of the immune system. The last effect results in immunosuppression and risk of common bacterial or viral infections [14]. Additionally, resistance may appear in some cases [15,16]. The absence of selectivity is determined by the mechanism of action, which is based on the disturbance of cell metabolism by blocking DNA syntheses and the cell mitosis. The adverse effects of the chemotherapeutics can be diminished by using drug delivery systems to increase the local concentration of anticancer molecules in cancer tissue and its cells [17,18]. Therefore, various types of chemotherapeutic carriers can be used: nanoparticles [19], hydrogels [20,21], composed nanoparticle–hydrogels [22], micelles [23], and liposomes [24].

Selectivity can be achieved by two approaches. The first one involves using composite particles bearing molecules that can specifically associate to the cell receptors overexpressed on the cytoplasmic membrane of cancer cells. However, this approach offers limited selectivity because such receptors are expressed also on the normal cells. The second approach is based on the possibility of the cancer cells to uptake colloid particles with submicron size by phagocytoses, while normal cells, except those of the immune system, lack this ability due to process of differentiation. Both approaches have the imperfection that the molecules of the anticancer chemotherapeutics attack normal cells as well as cancer cells.

### 3.2. Apoptosis

It is possible to avoid the use of chemotherapeutics through a principally different approach, which is based on the activation of apoptosis [25,26]. Apoptosis, called “programmed cell death”, is a genetically programmed intracell mechanism for the suicide of damaged or improperly functioning cells, as well as normal cells of the macroorganism undergoing hormonal involution in certain organs [27] and during maturation of the immune system [28]. Developed in the process of biological evolution, apoptosis occurs without toxic effects, in contrast with necrosis. This cell suicide program functions as an enzyme cascade of step-by-step activation of proenzymes into their active forms, ultimately resulting in the complete destruction of the cell into small molecules then used for the synthesis of new macromolecules in healthy cells [4,29]. Caspases (proteolytic enzymes that cut the polypeptide chains between cysteine and aspartate residues) play a key role in the process of apoptosis [30,31].

Apoptosis can be activated by two pathways: The extrinsic one starts with the binding of “tumor necrosis factor” to the extracellular domain of a “dead receptor”, whose intracellular domain activates procaspase-8 and procaspase-10. The internal pathway begins with a disturbance to the balance between the pro-apoptotic proteins (Bax and Bak) and anti-apoptotic proteins (Bcl-2, for example), which can be caused by damage factors such as radiation, free radicals, cytotoxic agents, viruses, tumors, etc. [32,33,34,35]. This leads to exit of the mitochondrial protein cytochrome *c* (cytC) from mitochondria into the cytoplasm. In the cytoplasm, cytC forms a complex (apoptosome) with the large molecular protein Apaf-1 (apoptosis activating factor), which acquire enzyme activity and activate procaspase-9, whose active form caspase-9 then activates procaspase-3 and procaspase-7. To activate the caspase cascade by the internal pathway, the concentration of the cytC-Apaf-1 apoptosome must reach some critical level at which the anti- and pro-apoptosis equilibrium shifts to the latter by overcoming a cytoplasmic cytC concentration threshold. Then, the two pathways converge at caspase-3 and caspase-7, which destroy the proteins of the cell.

### 3.3. Cytochrome c

**Figure 7:**
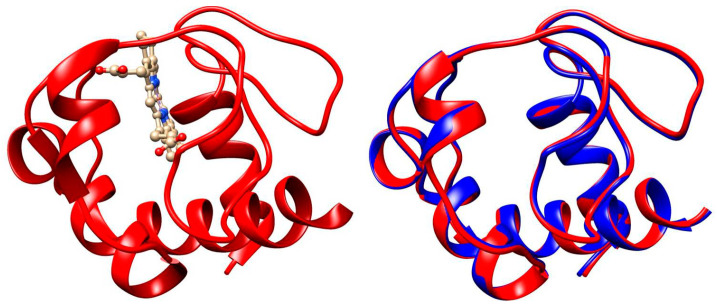
Three-dimensional structures of equine cytC (left model, PDB: 1HRC) and 3D aliment of equine (1HRC, red-colored patches) and human (3ZCF, blue) cytC apoproteins. The atomic coordinates correspond to the crystallographic structures deposed in the Protein Data Bank. The α-helix segments of the polypeptide chains are depicted as ribbons. In the right model, the heme is not shown.

Cytochrome *c* (cytC) is a small hemoproteid with a molecular mass of 12.4 kg/mol, composed of 104 amino acid residues and a flat heme heterocycle with a coordinately bound divalent iron (Fe^2+^) ion in the center. Its polypeptide chain, consisting of three α-helix segments, is coiled around the covalently bound heme cycle [36], forming a water-soluble globule 3 nm in size (Figure 7). The chargeable amino acid residues, located on the surface of the globule, determine its isoelectric point pI 9.3 [6,7]. At neutral pH, the net charge is positive due to predominance of the protonated groups of lysine, arginine, and histidine over deprotonated carboxylic groups of aspartate and glutamate amino acid residues. Because of its structure (short polypeptide chain coiled around the heme and covalently bound to it), the 3D conformation of the cytC globule is stable in a wide 3–12 pH range [37]. Acid or alkaline denaturation emerges at the extremes of pH ≤ 2 and pH ≥ 12 [38,39,40,41]. The cytC macromolecules retain their 3D structure and electron transport function when adsorbed on solid dielectric and metal surfaces, as proved by different techniques: surface-enhanced Raman scattering [42], electron spin resonance combined with UV-vis absorption spectroscopy [43], and electrochemical measurements (cytC retains its oxidation/deoxidation capability) [44,45,46,47,48].

In eukaryotic biological cells, cytC globules are associated with the electron transport protein complex incorporated in the internal mitochondrial membrane, where cytC works as an electron carrier due to Fe^2+^/Fe^3+^ oxidation/reduction of the ferro/ferry ion in the heme. Additionally, cytC plays a key role in the internal pathway of apoptosis, starting a multistage cascade of irreversibly biochemical reactions, resulting in complete destruction of the cells (Section 1.1). In cancer cells, however, apoptosis is blocked because their metabolism is based on the anaerobic utilization of glucose, leading to the impossibility of cytC exiting from the mitochondria [49,50,51]. Apoptosis can be initiated by introducing of exogenous cytC into the cytoplasm by microinjection [52,53,54]. Direct application of a cytC solution is ineffective because its hydrophilic globules cannot penetrate the hydrophobic bilayer of the cytoplasmic membrane of the cells. This difficulty can be overcome by the ability of cancer cells to phagocytize colloid particles with submicron size. For this purpose, we use equine cytC (extracted from a heart of a horse) because of its low cost (in difference from human cytC), which is allowed for application in human medicine. The alignment analysis reveals that the 3D structure of equine and human cytC is almost the same with 97% identity (Figure 7). The high degree of structural identity and the low molecular mass of the cytC macromolecule strongly decrease the probability of undesirable immune reactions during the in vivo application of equine cytC.

### 3.4. Cytochrome c Bearing Particles

To reach high anticancer cytotoxic efficiency using cytC-bearing colloid particles, it is required to satisfy the conditions formulated in Reference [9]. One of these is that the composite particles must be positively charged taking into consideration that phagocytosis begins with adsorption of a colloid particle on the negatively charged cytoplasmic membrane of cancer cells. Biological membranes are negatively charged due to predominance of acid lipids, whose content is about 10–20% in normal cells [55]. The external surface of the cytoplasmic membrane of cancer cells is even more negatively charged owing to the increased content of phosphatidylserine: approximately 20% compared to 5% in normal cells [56]. On the contrary, the bare colloid particles must be negatively charged to condition electrostatic adsorption of cytC macromolecules, which are positively charged at pH ≤ 9 [6,7].

On the other hand, a large number of cytC macromolecules need to be introduced during phagocytosis of a single cytC-bearing particle to overcome the cytC intracellular concentration threshold, estimated as 16 μmol cytC in 150 mM KCl [57], at which the process of apoptosis starts. This requires the use of larger particles, although their maximal size is limited because of the impossibility of the cell membrane to envelop a large particle. The optimal particle size for phagocytoses is about half a micrometer [58,59], considering that the lower size is also limited owing to the inability of the membrane to bend with a small radius.

The size limitation suggests the use of submicrometric colloid particles with a high effective adsorption area, such as porous particles. It seems that the number of cytC globules adsorbed onto one porous particle should be very high, taking into account their large internal surface area. However, experiments with mesoporous silica do not confirm the expectation for high cytotoxicity, although the mean pore diameter is several times larger than the 3 nm cytC globules [60,61,62,63,64]. In fact, cancer cell viability is comparable to that when using non-porous particles bearing cytC. It seems that the total surface area (measured by low temperature N_2_ adsorption) is not appropriate for estimation the adsorption capacity in the case of proteins, even when their globules are much smaller than the diameter of the pores. Our explanation is that the cytC globules cannot move into the particle interior because they cannot overcome the electrostatic repulsion of protein globules adsorbed on the porous entrance. I.e., the superficially adsorbed cytC globules create an electrostatic barrier that hinders the introduction of new protein globules into the pores.

We apply just the opposite approach using particles with a smooth surface whose effective area is equal to the geometrical one. For the purpose, we use the clay mineral montmorillonite, whose plate-like colloid particles have a high size/thickness ratio and pH-independent negative electric charge, which conditions the electrostatic adsorption of the positively charged cytC globules [65]. The smooth surface allows for easy and quick adsorption during the preparation of the cytC-bearing composite particles and desorption in cancer cells after their uptake. Probably, the rate of cytC desorption is an important factor in reaching the critical concentration of cytC-Apaf-1, which initiates the caspase cascade, considering the opposite process of destroying of this enzyme-active protein complex.

### 3.5. Cytotoxicity

The use of phagocytosis in colloid particles to introduce exogenous cytC into cancer cells allows for achieving both cytotoxicity and selectivity of the anticancer effect without using chemotherapeutics. In the literature, there are two decades of articles describing different cytC-bearing particles that cause a cytotoxic effect investigated in in vitro and in vivo experiments. Most authors reported maximal cytotoxicity, which does not exceed 60–80% using various sophisticated artificial nanoparticles bearing cytC, including solid-state dielectric as silica [63,65], hybrid gold–silica [66] and gold–ferric oxide even when cytC is combined with anticancer chemotherapeutics [67,68]. Higher cytotoxicity (up to 90%) is reached by using colloid particles prepared with cytC [69,70]. We achieved 97% cytotoxicity (ratio of dead to alive cells) in the 96th hour after addition of a suspension of the cytC-MM hybrid particles to a colon cancer cell culture [9,71] using montmorillonite colloid particles as a carrier of native cytC globules.

### 3.6. Montmorillonite

**Figure 8:**
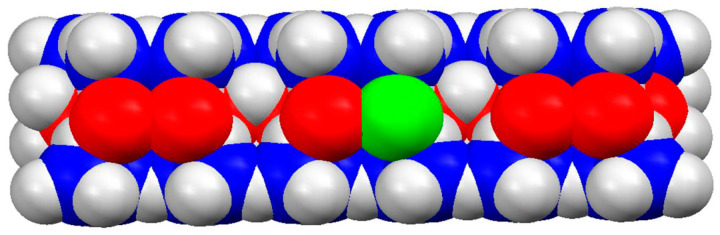
Model (fragment) of MM nanoplate with 0.9 nm thickness (the vertical size), generated by three elementary cells according to the atomic coordinates of its crystallographic structure. The trivalent aluminum atoms in the central sheet are colored in red. The green atom is bivalent (Fe^2+^ or Zn^2+^) extrinsic atom incorporated in the aluminum oxide sheet. The four-valent silicon atoms (blue colored) in the two lateral oxide sheets are partially covered by oxygen atom (not colored).

Montmorillonite (MM) is a plate-like alumosilicate clay mineral whose symmetrical crystal lattice has three sublayers (atom sheets): a central alumina sheet (AlO, Al^3+^ in octahedral coordination with O^2−^) and two lateral silica sheets (SiO, Si^4+^ in tetrahedral coordination with O^2−^) (Figure 8). This structure determines the flat form of MM nanoplates with a smooth surface, 0.9 nm thickness and large (submetric) size. MM colloid particles, obtained by excavation and successive rough and fine mechanical crushing and size screening, are packs (tactoids) consisting of several decades of monoplates with submicrometric to micrometric size [72,73]. Each monoplate has a pH-independent electric charge originating from extrinsic bivalent metal ions (Zn^2+^ and Fe^2+^ instead Al^3+^) incorporated by isomorphic exchange in the octahedral sites of the central alumina sheet and by partial substitution of Si^4+^ with Al^3+^ atoms in the tetrahedral sites of the two silica lateral sheets. The lower valency of the substituting atoms compared to the atoms with higher valency leads to the emergence of a negative effective charge.

In the tactoid, the negative charge of the monoplates is compensated by bivalent counterions such as Ca^2+^ cations located in the gaps between them. Therefore, van der Waals attraction between the monoplates prevails over the electrostatic repulsion and the tactoids are stable. Tactoids can be split down into monoplates by high-temperature boiling in saturated HCl acid. Then the replacement of the bivalent Ca^2+^ counterions with monovalent H^+^ leads to increased electrostatic repulsion, which prevails over the van der Waals attraction [74,75]. The final substance is obtained by neutralization by a monovalent base, as NaOH or KOH. The used by us commercial Na-montmorillonite K10, after solving in an aqueous medium, forms a colloid suspension consisting of monoplates with 1 nm thickness and submicrometric size. MM monoplates can be defined as nanoparticles (or nanoplates, due to their nanometric thickness), or as colloid particles (owing to their submicrometric size). In this article, we use three terms, monoplates, nanoplates, and microplates, depending on the context in which the particle dimensions determine a given property.

Due to their extremely small thickness, MM nanoplates have a very high surface/mass ratio (about 250 m^2^/g, measured by low-temperature N_2_ adsorption) and consequently, high adsorption capability. A second peculiarity of MM nanoplates is their negative surface electrostatic potential (created by the effective charge of the crystal lattice), due to which MM plates can be used as a substrate for electrostatic adsorption of positively charged small and high-molecular substances such as polymers and proteins. The huge size/thickness ratio allows for investigating the adsorption of protein macromolecules by measuring the mass inclement of MM monoplates by static light scattering [76].

### 3.7. Cytochrome–Montmorillonite Hybrid Particles

To compose cytC-MM particles with optimal size for phagocytosis, we used an aqueous suspension of MM colloid monoplates with half-micrometric size, obtained by differential centrifugation [11]. We then added a cytC solution at neutral pH, where the net charge of the cytC globules is positive (Figure 1). Under these conditions, electrostatic adsorption of cytC onto MM monoplates occurs due to their opposite electric charges. The adsorption is manifested by the decrease in the negative charge of MM nanoplates, as demonstrated by the electrophoretic mobility of cytC-MM particles (curve 3 in Figure 5), measured by microelectrophoresis (Section 2.3). To avoid particle aggregation (and by that to retain their size unchanged) we use cytC concentrations different from those at which the total charge of the cytC-MM microplates is close to zero (point of recharging), when the electrostatic repulsion is weaker that the van der Waals attraction between the neighbor cytC-MM colloid plates. With an excess of cytC in the suspension, saturated adsorption occurs, causing the total charge of cytC-MM particles to reverse its sign from negative to positive because of the presence of protein globules on the MM surface. Due to the over-equivalent adsorption, cytC-MM monoplates obtain a positive net charge (Figure 3), which prevents their aggregation and conditions their electrostatic adsorption onto the negatively charged cytoplasmic membrane of cancer cells. In the process of recharging, a temporary state with zero charge emerges, potentially leading to aggregation because of the van der Waals attraction between the cytC-MM monoplates. Nevertheless, aggregation does not appear because the protein adsorption is much faster than particle collision due to the significant difference in diffusion coefficients (determined by particle size and viscosity of the medium) between nanometric cytC globules and submicrometric MM monoplates. Our results on cytC adsorption onto MM nanoplates disclose that at saturated adsorption, cytC globules form a protein monolayer with density of 8 cytC globules per 100 nm^2^, occupying about two-thirds of the MM surface [8].

### 3.8. Adsorption or Aggregation

Equation (1) describes the increment Δ*R*_θ_ in the light scattering coefficient *R*_θ_ (measured at the scattering angle θ), which reflects the increment Δ*M* in the mass *M* of the MM nanoplates in two modes: (a) The dependence *R*_θ_~*N*_0_*M*^2^ (the mass *M* is squared) describes the light scattering increase at constant particle concentration *N*_0_ (the number *N* of free MM and cytC-MM plates in a unit volume). Then, the mass *M* grows due to the adsorption of cytC globules on MM nanoplates. (b) The dependence is linear according to the particle mass—*R*_0_~*cM* when the mass grows as a result of aggregation of cytC-MM nanoplates—because then the number *N*_0_ of free MM particles decreases. The difference in the two modes of Equation (1) comes from the fact that the light scattering in an aqueous suspension, containing MM and cytC, is almost entirely caused by the MM and cytC-MM colloid particles, but the contribution of the free protein globules is omissible. This reflects the drastic difference in the mass of a single MM nanoplate and a single cytC macromolecule. In the experiments described below, the weight concentration c of MM nanoplates remains constant. So, only the mass *M* of cytC-MM particles and the particle concentration *N*_0_ = *cN*_A_/*M* (in units [L^−1^], where *N*_A_ [mol^−1^] is the Avogadro’s number) can undergo alterations. The weight concentration *c* = 3 mg/L MM of the colloid particles in the suspension was chosen to be low enough to avoid the cytC-MM particles aggregation in the moment of their recharging from negative to positive total charge (Section 3.7).

To distinguish between the two modes of Equation (1) and to interpret the mass increment Δ*M* entirely as a result of the protein adsorption, it is necessary to exclude the cytC-MM particle aggregation as a possible cause for the increase in the mass *M*. For that, an additional criterion is needed. As an indicator for aggregation, we use the relaxation time τ of the electrooptical effect (Equation (2) in Section 4.2) after field switching off. The velocity of particle disorientation is then determined by the size *B* of the particles in the suspension and the viscosity of the medium (Equation (3)). Due to the cubic dependence τ~*B*^3^, the relaxation time τ is very sensitive quantity to the particle size *B*. When flat particles such as cytC-MM nanoplates aggregate face-to-face, the common surface of the aggregate grows because the new plate only partially covers the surface of the aggregate, the rest of its surface remains free and sticks up in the medium. This causes additional friction at aggregate moving due to the viscosity of the liquid. Therefore, the disorientation of MM and cytC-MM plates after switching off the electric field becomes slower and the relaxation time increases. This is equivalent to an effective growth in the particle size *B*, which leads to a strong increase in the relaxation time τ, although in this case, the dependence τ(*B*) is not cubic. Our previous investigations showed that τ increases drastically when the concentration of cytC in MM suspension is in the range of particle recharging (the change of the net charge of cytC-MM plates form negative to positive), which appear at 5:3 mg/mg cytC/MM ratio [7,11]. The aggregation does not emerge in the low and high ratio of the weight concentrations of cytC and MM when the electrostatic repulsion prevails over the van der Waals attraction. In the present investigation, only concentrations of cytC and MM are used at which the cytC-MM suspension is stable.

### 3.9. Cooperative Effect

The density of the protein layer on a solid surface (number of protein globules per unit surface area) is determined by the size of the globules and the intermolecular interactions between them. When there is enough free area (low degree of surface occupation), protein globules can be adsorbed independently (absence of intermolecular interactions), or dependently when newly adsorbed globules are influenced by those already adsorbed. In the last case, a cooperative effect arises, which can be either negative or positive depending on the balance between the electrostatic repulsion among identically charged protein globules and the van der Waals attraction between them.

The intermolecular interactions between protein globules adsorbed on a solid surface are the object of the present investigation. For this, we have chosen the globular protein cytochrome *c* (cytC), which has very stable 3D structure (Section 3.3), and the clay mineral montmorillonite (MM) in monoplate form (Section 3.6), whose extremely thin monoplates allow for measuring the mass increment Δ*M* at adsorption of three times bigger cytC globules. This research aims to find out whether cytC globules are adsorbed independently or if a cooperative effect appears: negative (caused by the predominance of electrostatic repulsion over the van der Waals attraction), or positive in the opposite case. Therefore, we investigated the adsorption isotherm (dependence of the adsorbed adsorbate on its concentration at constant temperature) using the relative mass increment Δ*M*/*M*_0_ as a criterion, which grows from zero to a saturated value as the cytC concentration *C*_cyt_ in the suspension of the MM monoplates increases. Taking into consideration that the adsorption is temperature- and pH-dependent, we measure the adsorption at constant (room) temperature and pH 6.5, where cytC globules are charged oppositely to the MM surface (Figure 3).

The protein adsorption isotherm can have three forms: linear, convex, and concave. The form of the adsorption isotherm depends on the intermolecular distance, which decreases with the degree of surface occupation. (a) A liner Δ*M*/*M*_0_ = *f*(*C*_cyt_) dependence of the relative mass increment on cyC concentration is expected when the interaction between the protein globules is negligible; such a situation appears at a low degree of surface occupation. Then, every approaching cytC globule can be adsorbed if it finds enough free spot on the MM surface. This concentration range can be somewhat extended by adding an indifferent salt whose counterions weaken the electrostatic repulsion between the cytC globules. (b) Δ*M*/*M*_0_ = *f*(*C*_cyt_) dependence with a decreasing slope (convex curve) appears at strong repulsion between the similarly charged protein globules at a higher degree of surface occupation and low ionic strength of the medium. Then, the intermolecular electrostatic repulsion hinders the adsorption of new globules (negative cooperative effect). Such dependence seems most probable in our experiment because cytC globules are positively charged at pH 6.5 and there is no added salt in the suspension. (c) Δ*M*/*M*_0_ = *f*(*C*_cyt_) dependence with an increasing slope (concave curve) indicates a positive cooperative effect, which can appear when specific (hydrogen bonds) and non-specific (van der Waals) attractive forces between the protein globules predominate over the electrostatic repulsion caused by their positive net charge. The experiment discloses (Figure 5, curve 1) that, contrary to the expectation, this third possibility takes place at adsorption of cytC on the MM nanoplates.

In the literature, three types of protein adsorption are defined: non-cooperative, positive cooperative, or negative [77,78]. A negative cooperative effect appears because of the electrostatic repulsion between the neighboring protein globules whose net electric charge is identical. A positive cooperative effect emerges in the isoelectric point due to absence of intermolecular electrostatic repulsion. In the present research, we reveal that a positive cooperative effect can arise even when the protein globules are electrically charged (positively at pH 6 in the case of cytC) due to the irregular distribution of the Coulombic charges on their surface, which creates a patch-like local electrostatic potential with opposite sign in different areas (Figure 2). This conditions the mutual orientation of the protein globules, like dipole–dipole one, due to the minus to plus attraction (Figure 6). Therefore, the possibility of positive or negative cooperative effects depends on the individual structure of the given globular protein, pH, and the ionic strength of the medium (because the counterion concentration determines the effective electrostatic forces between the charged globules by shielding of the surface potential). An indication of a positive cooperative effect is the observed exponential time-dependent adsorption with minute kinetics of cytC on a negatively charged surface modified by covalently bound COOH groups [79].

### 3.10. Protein Adsorption

The adsorption of the water-soluble protein cytochrome (cytC) on the surface of montmorillonite (MM) clay nanoplates is electrostatically conditioned by the opposite electric potential of the cytC adsorbate and the MM adsorbent: the net charge of cytC positive is positive at pH 6.5 (Figure 1), and the pH-independent charge of MM is negative. The adsorption is over-equivalent: the electrophoretic mobility of the cytC-MM particles reveals the recharging of MM plates from negative to positive (Figure 3). The isoelectric point of cytC-MM particles divides the protein concentration dependence into two ranges: lower (≤3:3 mg/mg cytC/MM ratio) and upper (≥10:3 cytC/MM), in which particle aggregation does not appear (Figure 4). In the two concentration ranges, the mass of MM plates only grows because of cytC adsorption. The saturation of the cytC adsorption (curve 2 in Figure 5) allows for choosing 10:3 mg/mg cytC/MM as the optimal ratio at which a cytC-MM hybrid particle delivers the maximum cytC globules into a cancer cell upon its uptake [9]. The chosen pH 6.5 is suitable for adsorption of cytC on MM and it is close to the optimal pH 6.7–7.1 for the cancer cell culture [80]. This allows for avoiding the use of additional buffer, which could be toxic to the cells.

The over-equivalent electrostatic adsorption (recharging, Figure 3) can be caused by additional (non-electrostatic) protein–surface attraction or by protein–protein association. The first type of interaction appears in a clear form at a low degree of surface occupation by the protein globules, and the second type—at a high degree of occupation, respectively—at long or short intermolecular distance. The mean distance between neighboring protein globules drastically decreases during adsorption on solid/liquid interface (surface-induced concentrating), compared to the case of protein solution at the same volume concentration. So, to distinguish the two causes of electrostatic recharging, it is necessary to investigate the protein concentration dependence of adsorption using different physical quantities. We chose the mass *M* and electrophoretic mobility μ (mechanical and electric properties), employing appropriate methods: static light scattering and microelectrophoresis (Figure 5).

### 3.11. Mass Growing

As an indicator for adsorption of cytC globules on MM plates, we use the mass increment Δ*M* of cytC-MM particles. This opportunity is determined by the flat form which results in a high surface/mass ratio (in difference from the spherical form) and very small (1 nm) thickness of MM nanoplates. At saturated bilateral adsorption of the three times bigger cytC globules, the thickness of cytC-MM hybrid particles reaches 7 nm, and the measured light scattering intensity *I*_0_ increases twofold (Figure 4, Insert). This allows for experimentally determining the mass increment Δ*M* employing Equation (1).

However, this approach is complicated by the possible aggregation of cytC-MM. Then, Δ*M* could have two components: protein adsorption and particle aggregation. In the lower *C*_cyt_ range, aggregation can arise because of the reduction in the electrostatic repulsion between neighboring cytC-MM particles when cytC concentration approaches the recharging point, which appears at a 5:3 mg/mg cytC/MM ratio [11]. In the higher *C*_cyt_ range, the aggregation could also be possible because every cytC-MM particle passes through a transient isoelectric state in the process of its recharging. To avoid the aggregation, we use a diluted suspension (3 mg/L MM) in which the mean distance between the cytC-MM particles is long enough and a cytC/MM ratio (10:3 mg/mg) at which the MM plates are completely recharged.

To verify the absence of aggregates, we studied the relaxation process Δ*I*_t_(*t*) of the electrooptical effect with the time *t* after switching off the field (Equation (2)), which is very sensitive due to the strong dependence τ~*B*^3^ (Equation (3)) of the relaxation time τ on the size *B* (the function is cubic when the particles are unaltered; in the case of face-to-face disks aggregation, the dependence is weaker). The experiments showed that the values of τ increase strongly in the vicinity of the isoelectric point [7], but aggregation is absent in the lower and upper cytC concentration ranges (Figure 4).

### 3.12. Structure of the Adsorption Layer

Two principal models of protein association on a solid surface can be supposed: horizontal and vertical. In horizontal association, the cytC globules lie directly on the surface until they form a complete monolayer. In vertical association, an approaching globule lies on the already-formed protein monolayer and forms a second one. However, the absence of a stair in the increment curve (Δ*M*/*M*_0_)(*C*_cyt_) at saturated adsorption (line 2 in Figure 5) disproves the latter supposition. It is reasonable to predict a mixed variant of surface protein aggregation: the approaching cytC globule lies vertically on monolayer protein islands (positively charged protein patches on the negative MM surface), which are already formed by horizontal protein aggregation around the adsorbed single cytC globules.

Measuring the mass increment Δ*M* at protein adsorption does not allow for differentiation between the possible types of protein association because it reflects the total amount of adsorbed protein. This is why we employed microelectrophoresis as a complementary method that provides information on the protein occupation of the particle surface. This approach is based on the decrease in the negative potential of MM nanoplates at adsorption of the positively charged cytC globules, and the found coincidence of the isoelectric point of cytC-MM particles (at saturated adsorption) with that of free cytC globules [6,7]. The last fact means that (a) the electrophoretic mobility of cytC-MM particles is determined only by the protein layer and does not sense the surface charge of MM surface; (b) the initially adsorbed cytC globules are arbitrarily oriented, and the averaged orientation of all globules in the monolayer is random. This allows for the use of the electrophoretic mobility μ as an indicator for the degree of occupation of the nanoplate surface.

The electrophoretic mobility is a function of the zeta-potential ζ, according to Equation (4). However, this equation is valid for colloid particles with smooth surfaces. When a globular protein is adsorbed on such particles and the protein globules form a mono- or multilayer at saturated adsorption, the electrophoretic mobility μ is determined completely by the charge of the protein globules because the particle surface is hydrodynamically shielded. When the adsorption is incomplete, the zeta-potential is not proportional to the surface charge density because the friction of the adsorbed protein globules is higher than that of the smooth surface of the colloid particle. Therefore, we use the measured electrophoretic mobility μ instead of the calculated electrokinetic potential ζ usually given in the biological literature.

Comparing the protein concentration dependences of the electrophoretic mobility μ(*C*_cyt_) and the mass increment Δ*M*(*C*_cyt_) allows for distinguishing between vertical and horizontal association of cytC globules in the protein layer on the MM surface. The divergent course of the two concentration dependences (curves 1 and 3 in Figure 5), together with the absence of a stair in line 2, allows for us to conclude that the cytC globules form a quasi-monolayer with a mixed horizontal/vertical association of protein globules.

### 3.13. Protein Docking

To calculate the association energy, we developed a computer procedure, briefly described here. As the first step, we calculated the surface electrostatic potential of a single cytC globule by the program for protein electrostatics Propka (versions: PDB2PQR 3.6.1 and APBS 3.4.1), using 3D crystallographic atomic coordinates of equine cytC, taken from the Protein Data Bank (PDB:1HRC), and then visualized the potential according to its value and sign (Figure 2). In the second stage, we generated 3D atomic coordinates of the cytC dimer (Figure 6) by the program for rigid protein docking pyDockWEB (https://life.bsc.es/pid/pydockweb, accessed on 6 May 2024) [81] in the mode of preferred orientation of the two globules, so that the negatively charged surface area of the first cytC globule contacts with positively charged surface of the second one.

The number of possible models with such electrostatically preferred orientation is relatively small because at pH 6.5 the local electrostatic potential on the surface of the cytC globule is predominately positive, whereas the negatively charged surface areas are only two: one bigger with higher potential and one smaller with lower potential (Figure 2). The number of possible models increases if we satisfy the second condition for energetically advantageous orientation: complementary relief of the contacting surfaces. The previous (preferred) mutual orientation of the two globules was chosen assuming that the most stable dimer should be the one in which the bigger negatively charged area of the first globule contacts with the strongest positively charged surface of the second one, and the contacting surfaces are geometrically complementary. This expectation is confirmed by the results in Table 1: the more compact dimer-2 with strongly charged contact surfaces is much more stable in comparison with the less compact dimer-1. The geometrical complementarity is indicated by the value of the van der Waals component of the association energy Δ*G*_assoc_: its higher absolute value means stronger attraction due to bigger number of atoms located at a short distance in the contact region. Nevertheless, the electrostatic attraction gives the main contribution to the Δ*G*_assoc_: the dimer-1 with weaker charged contact surfaces is less thermodynamically stable (Table 1).

The docking program generates exact 3D atomic coordinates of dimers, giving a list of approximately one hundred models with decreasing absolute value |−Δ*G*_assoc_| of the association energy, beginning with the model with minimal free energy (maximal stability), estimated mainly by the van der Waals component Δ*G*_Waals_ of Δ*G*_assoc_. In the third stage, we computed the electrostatic component Δ*G*_electric_ of the first ten models, employing the program for protein electrostatics Bluues-Single (v. 2.0) [82,83]. As the most thermodynamically stable dimer, we selected the model with the maximal sum of the two components, |−Δ*G*_assoc_| = Δ*G*_Waals_ + Δ*G*_electric_, taking the last quantity from the docking program for the chosen model. The sign of Δ*G*_Waals_ is always negative (van der Waals attraction), and the sign of Δ*G*_electric_ can be positive (electrostatic repulsion) or negative (attraction), depending on the sign of the electrostatic potentials of the contacting surfaces of the two protein globules.

To construct the cytC trimer, we repeated the above procedure, assuming that the dimer acts as a rigid (fixed 3D atomic coordinates) protein globule with two identical domains. Again, as a first step, the surface electrostatic potential of the chosen dimer was computed by the program Propka. Then, the cytC trimer was constructed by docking of the dimer and a single cytC globule with the preferred orientation of the third cytC globule with its bigger negatively charged surface area to the strongly positively charged surface of one of the dimer’s globules. The 3D atomic coordinates of the dimer (and its two monomers) remain unchanged at association of the third cytC globule (rigid docking); the trimer is considered as a rigid protein globule with three identical domains. Then, the electrostatic component Δ*G*_electric_ of the trimer’s association energy Δ*G*_assoc_ was calculated by the program for protein electrostatics, Bluues, for the first ten models, generated by the docking program pyDockWEB; the latter gives the van der Waals component Δ*G*_Waals_. The most thermodynamically stable cytC trimer-1 and trimer-2, described in Section 2.5, were selected from these decade of models, using as a criterion the maximal absolute value of the association energy |−Δ*G*_assoc_| (minimal free energy), calculated as a sum of the electrostatic and van der Waals components. In the most energetically advantageous trimer-2, the third cytC globule contacts with the two others, and all three monomers form a dense trimeric associate (Figure 6). At this mutual position, both electrostatic and van der Waals components have maximal absolute (negative by sign) values (Table 1), i.e., the trimer is formed by maximally attractive forces.

### 3.14. Folding Energy

The electrostatic component of the association energy Δ*G*_electric_ = Δ*G*_N-mer_ − *N* × Δ*G*_mono_ was calculated as a difference between the folding energies Δ*G*_fold_ of the *N*-mer associate and *N* number monomers (*N* = 2 for dimer, *N* = 3 for trimer). The associate is considered as a globular protein with a united polypeptide chain, forming two or three rigidly bound identical domains. The negative value of the folding energy Δ*G*_fold_ predicts 3D structural stability, determined by four components: hydrophobic (hydration energy), electrostatic (attraction and repulsion between the Coulombic charges), van der Waals attraction (determined mainly by London dispersion forces) and intramolecular hydrogen bonds of the segments with α-helix and β-sheet structures.

To calculate the electrostatic (pH-dependent) component of the folding energy Δ*G*_fold_ of the cytC monomers, dimers, and trimers, we used the Bluues computer program for protein electrostatic calculations [83,84], which calculates the decrement −Δ*G*_fold_ in the Gibbs free energy at an imaginary conformational transition of the polypeptide chain from a random coil (the intramolecular forces are ignored) to a protein globule with fixed 3D atomic coordinates (the electrostatic interactions between all Coulombic charges are calculated). The pH-dependent (electrostatic) component of the folding energy of cytC dimers and trimers with different values of the electrostatic potential in the oppositely charged contact surfaces and with different geometrical complementation, described in Section 2.5 and Section 3.13), named here as Model-1 and Model-2, are given in Table 2.

For verification of the calculations, we calculated the electrostatic component of the folding energy Δ*G*_fold_ of the polypeptide chain of a single cytC apoprotein, using 3D coordinates of the crystallographic structure PDB: 1HRC of equine cytC protein. At pH 6.5, the computed values are Δ*G*_fold_ = −59 kJ/mol at ionic strengths 0.05 mol/L (used in the calorimetric experiments) and Δ*G*_fold_ = −42 kJ/mol at 0.0001 mol/L (the ionic strength in our experiments). These values are in satisfactory agreement (by the absolute values) with the free energy of unfolding Δ*G*_unfold_ = +(40–70) kJ/mol, measured by different experimental methods: differential calorimetry [84,85], optical [86], and a combination of both techniques [87].

### 3.15. Association Energy

At association of two or more protein globules with stable 3D structures, only the electrostatic and van der Waals components of the association energy Δ*G*_assoc_ change, whereas the hydration energy and the hydrogen bonds remain unaltered. We disregard the probable dehydration energy in the formation of cytC associates because the strongly bound water molecules remain on the hydrophilic surfaces of the globules in the contact areas, as the X-ray analysis of cytC crystal discloses. The maximal absolute value of the sum of the electrostatic and van der Waals components of the association energy Δ*G*_assoc_, computed by the programs Bluues and pyDockWEB, respectively, (Section 3.13 and Section 3.14) was used to select the dimers and trimers with maximally stable 3D structure.

The results in Table 1 for the cytC dimer with maximally dense packing (Model-2, shown in Figure 6), disclose that the electrostatic component Δ*G*_electric_ gives the main contribution: 92% of the total association energy Δ*G*_assoc_, under the experimental conditions applied at adsorption of cytC on MM nanoplates: pH 6.5 in aqueous medium with ionic strength 0.0001 mol/L (almost pure water). In the case of a thermodynamically less stable and less dense cytC dimer (Model-1), also constructed by negative-to-positive orientation of the charged surfaces, the electrostatic component is even 95%, although both components of the association energy are smaller. These results are in agreement with the literature data showing that the value of the van der Waals component is less than 10% of the total association energy [88] of protein dimers. This ratio allows for considering the electrostatic interactions between neighboring cytC globules (dependent on their mutual orientation) as the main factor leading to the found positive cooperative effect at cytC adsorption on the solid surface of MM nanoplates (Section 2.4, Figure 5).

## 4. Materials and Methods

### 4.1. Materials

Na-montmorillonite K10, activated by acid treatment (Sigma-Aldrich, Darmstadt, Germany), was suspended in triple-distilled water, sonicated for 5 min at 20 kHz with ultrasonic disintegrator (Techpan, Warsaw, Poland), and a fraction with low polydispersity was obtained by fourfold centrifugation [11]. The concentration of the obtained MM suspension was determined by weighing the dry remainder after completed drying in a microwave oven. The aqueous solution of cytochrome *c* (cytC) (Calbiochem, San Diego, CA, USA, Equine heart, Cat. 250600) was prepared by dissolving the dry protein powder in triple-distilled water. The cytC-MM suspension was prepared with continuous stirring for 30 min at room temperature by slowly adding the protein solution to the MM suspension up to a final concentration 10 mg/L cytC in 3 mg/L MM. The pH of the suspension was adjusted to pH 6.5 by KOH.

### 4.2. Static and Electric Light Scattering

At chaotic orientation of colloid particles (MM or cytC-MM nanoplates) with molecular mass *M* and particle concentration *N*_0_ (number *N* of the particles in a unit volume of a diluted liquid suspension), the intensity *I*_0_ of the beam scattered at angle θ is proportional to intensity *I*_00_ of the exiting beam. The light scattering coefficient (Rayleigh ratio) *R*_θ_ [m^−1^] is determined by the weight concentration *c* [g/L] = *MN*_0_/*N*_A_ of the suspended substance in scattering volume Ω placed at a distance *r* from the photoreceiver [89]:

*R*_θ_ = (*I*_0_/*I*_00_)(*r*^2^/Ω) = [*HM*^2^*N*_0_*P*(θ)] = *cHMP*(θ), (1)

where the optical constant *H* is defined by the wavelength λ_0_ in vacuum and the difference Δ*n* = *n*_1_ − *n*_0_ in the refractive indexes of the particles *n*_1_ and the medium *n*_0_. The function of intraparticle light interference (form-factor) *P*(θ) at scattering angle θ is determined by the form and the relative size *B*/λ of particles with diameter *B* at wavelength λ = λ_0_/*n*_0_ in the medium.

The electric light scattering method is based on the electrooptical effect (EOE), which arises at orientation of colloid particles in electric field; then, the intensity *I* of the scattered beam increases from *I*_0_ to *I*_E_. The particle disorientation after switching off the field leads to the decay of EOE: the increment Δ*I* = *I*_E_ − *I*_0_ decreases with the time *t*:

Δ*I*_t_ = Δ*I*_s_ exp(−*t*/τ), (2)

where Δ*I*_t_ and Δ*I*_s_ are the moment and steady-state values of the EOE, respectively. The relaxation time τ (*B*, η, *T*) (defined as time for decrease in EOE down to 37% of Δ*I*_s_) is determined by the particle geometry (form and size *B*) ant the viscosity η of the liquid medium with temperature *T*. In aqueous suspension of disk-like particles with diameter *B* at *T* = 20 °C:

τ = (2η/9*kT*) *B*^3^ = 59 *B*^3^ (3)

where *k* is the Boltzmann constant and τ [ms] and *B* [μm] are given in milliseconds and micrometers, respectively [76].

To measure *I*_0_, *I*_00_, and the relaxation dependence Δ*I*_t_(*t*), a computerized home-made apparatus was used (described in Ref. [11]) at a scattering angle θ = 90°. A sinusoidal electric field with a frequency ν = 5 kHz and voltage up to 140 V, generated by a Wavetek-185 functional generator and amplified by a Krohn-Hite-7500 amplifier, was applied in the cytC-MM suspension using two horizontal platinum electrodes.

### 4.3. Microelectrophoresis

The velocity of migration of free colloid particles in a direct electric field is proportional to the field strength *E* and the surface density of their electric charges. The electrophoretic mobility μ = *v*/*E* (velocity *v* of a colloid particle in an electric field with strength *E*) is determined by their electrokinetic potential ζ, the bulk viscosity η (at temperature *T*), and the dielectric permittivity ε_0_ε of the medium. According to Smoluchowski–Hückel–Henry’s equation [90,91]:

μ = (εε_0_/η) *f*(*a*/δ) ζ, (4)

where the Henry’s function *f*(*a*/δ) (defined by the ratio *a*/δ of the radius *a* of a dielectric particle to the thickness δ of its electric double layer, EDL) can vary from 2/3 to 1, depending on the particle form and the ionic strength of the medium; *f*(*a*/δ) = 1 in Smoluchowski equation (thin EDL and ratio *a*/δ ≥ 100).

The velocity *v* = *l*/*t* (time *t* for which a particle migrates a fixed distance *l*) of MM or cytC-MM particles was measured in a closed vertical flat electrophoretic cell using a Mark II apparatus (Rank Brothers, Newbury, UK) with a dark-field microscope. The mean velocity *v* of 20 + 20 particles was measured at reversing the field direction for each particle and focusing on the two stationary planes where the electroosmotic flow is zero.

### 4.4. Computer Techniques

The following computer programs were used: (a) Propka [92,93] for protein electrostatics to calculate the surface electrostatic potential of cytC monomers, dimers, and trimers; (b) pyDockWEB for rigid protein docking [94] to generate models of cytC dimers and trimers; (c) Bluues [95] for protein electrostatics to calculate the electrostatic component of the folding energy of the cytC polypeptide chain in monomers, dimer, and trimer forms; (d) SuperPose (http://wishart.biology.ualberta.ca/SuperPose, accessed on 6 May 2024) for macromolecular alignment to compare the 3D structure of the equine and human cytC macromolecules; (e) Chimera (v. 1.15) [96], PBEQ Solver (https://charmm-gui.org/?doc=tutorial&project=pbeqsolver, accessed on 6 May 2024) [97], and VMD: Visual molecular dynamics 1.9.2 [98] for visualization of 3D models and the electrostatic potential on the surface of cytC monomers, dimers, and trimers. The atomic coordinates of the monomeric cytC and apo-cytC models correspond to the crystallographic structure of equine cytC with ID 1HRC in the Protein Data Bank. The model of MM nanoplate with atomic coordinates was generated and visualized by the program Mercury 3.8 using the crystallographic atomic coordinates of the elementary crystal cell source X-ray simulation structure from American Mineralogist Crystal Structure Database with code 0002868 [99].

## 5. Conclusions

The electrostatic adsorption of the positively charged at pH 6.5 globular protein cytochrome *c* (cytC) onto negatively charged colloid nanoplates of the alumosilicate mineral montmorillonite (MM) was experimentally investigated by the methods of static light scattering and microelectrophoresis. As expected, cytC globules are adsorbed independently at a low degree of surface occupation, when the intermolecular interactions between the new and already adsorbed protein globules are negligible. When the intermolecular distance decreases with the protein adsorption, it is reasonable to expect a negative cooperative effect because of the electrostatic repulsion between the positively charged cytC globules. However, the results disclose that a positive cooperative effect appears instead of the expected negative one when the protein globules occupy more than one third of the MM surface. This inference is based on the observed mass increment of cytC-MM nanoplates at increasing protein concentration and the parallel alteration in electrophoretic mobility (decreasing under the point of recharging and increasing above it) at steady-state adsorption.

To reveal the cause of the experimentally found cooperative effect, a computer procedure was developed, which allows for the quantitative estimation of the decrease in Gibbs free energy at the association of identical protein globules. The calculation of the association energy of cytC dimers and trimers reveals that the positive cooperative effect is caused by protein association due to the predominance of the electrostatic attraction over the repulsion between the identically charged cytC globules, which occurs at a specific mutual orientation of the neighboring cytC globules. This specific intermolecular attraction is conditioned by the irregular distribution of the charged amino acid residues, which leads to the appearance of a patch-like local electrostatic potential on the surface of the protein globules. At positive-to-positive orientation of the contact surfaces, a protein association is impossible, but at negative-to-positive orientation, the electrostatic attraction predominates and cytC globules form associates. The comparison of the two components of the association energy discloses that the electrostatic forces give the main contribution, while the van der Waals attraction plays a subsidiary role.

## Figures and Tables

**Table 1:** Association energy ΔGassoc [kJ/mol] of cytC dimers and trimers at pH 6.5, ionic strength 0.0001 mol/L and temperature 20 °C and its electrostatic and van der Waals components.

Associate	Electrostatic Energy	Van-Der-Waals Energy	Association Energy
Dimer-1	−221.3	−11.0	−232.3
Trimer-1	−319.2	−7.3	−326.5
Dimer-2	−326.0	−26.5	−352.5
Trimer-2	−523.9	−11.6	−535.5

**Table 2:** Electrostatic component of the folding energy Δ*G*_fold_ [kJ/mol] of cytC monomers, dimers, and trimers at pH 6.5, ionic strength 0.0001 mol/L, and temperature 20 °C.

Associate	Monomer	Dimer	Trimer
Model-1	−42	−306	−445
Model-2	−42	−410	−651

## Data Availability

The original contributions presented in this study are included in the article; further inquiries can be directed to the corresponding author.
